# Reviewer acknowledgement

**DOI:** 10.1186/1757-7241-22-4

**Published:** 2014-03-10

**Authors:** Hans Morten Lossius, Kjetil Søreide, Kristi G Bache

**Affiliations:** 1 Norwegian Air Ambulance Foundation, Drøbak, Norway; 2 Stavanger University Hospital, Stavanger, Norway

## Abstract

**Contributing reviewers:**

The *Scandinavian Journal of Trauma, Resuscitation and Emergency Medicine*—which received its first Impact Factor in 2013—is extremely grateful for the time, hard work and support of its highly-qualified peer reviewers. The editors of the *Scandinavian Journal of Trauma, Resuscitation and Emergency Medicine,* the Norwegian Air Ambulance Foundation and BioMed Central would like to show our appreciation by thanking the following people for their assistance reviewing manuscripts for the journal in 2013.

## 

Haakon Abrahamsen

Norway

Jose Adams

United States of America

Vanessa Agostini

Italy

Sara Ahmadi

United States of America

Shubha Allard

United Kingdom

Bahaaldin Alsoufi

United States of America

Claus Andersen

Denmark

Hagen Andruszkow

Germany

Per Åsheim

Norway

Alexandre Augusto

Brazil

David Baker

France

Matt Baker

United Kingdom

Jan – Bakker

Netherlands

Miklosh Bala

Israel

Zsolt Balogh

Australia

David Bar

United States of America

Greg Beilman

United States of America

Sigrid Beitland

Norway

Michael Bemelman

Netherlands

Jozsef Betlehem

Hungary

Joost Bierens

Netherlands

Ulf Björnstig

Sweden

Conrad Arnfinn Bjørshol

Norway

Antonio Blesa

Spain

Katarina Bohm

Sweden

Georg Bollig

Germany

Massimo Bonacchi

Italy

Mal Boyle

Australia

Mikkel Brabrand

Denmark

Dominik Brammen

Germany

Stevan Bruijns

United Kingdom

Pablo Cardinal

Uruguay

Maaret Castren

Sweden

Gianfranco Cervellin

Italy

Won Chul Cha

South Korea

Adam Chesters

United Kingdom

Arturo Chieregato

Italy

Chee Fah Chong

Taiwan

Erika Frischknecht Christensen

Denmark

John Clarke

United States of America

Tim Coats

United Kingdom

Jim Connolly

United Kingdom

D Jamie Cooper

Australia

Tobias Cronberg

Sweden

Andrew Dawson

Australia

Mohamud Daya

United States of America

Michel Debacker

Belgium

Trond Dehli

Norway

Bart Demaerschalk

United States of America

Cornelius Donat

Germany

Yingchun Dong

China

John Donnelly

United States of America

David Dries

United States of America

Francois Xavier Duchateau

France

Remco Ebben

Netherlands

Signy Camilla Eidstuen

Norway

Ulf Ekelund

Sweden

Sabina Fattah

Norway

Ole Magnus Filseth

Norway

Gerry FitzGerald

Australia

Arthas Flabouris

Australia

Lars Folkestad

Denmark

Knut Fredriksen

Norway

Hans Friberg

Sweden

Richard Galgon

United States of America

Miguel Galicia

Spain

Massimo Gallerani

Italy

Richard Gamelli

United States of America

Luis Garcia Castrillo

Spain

Sven Erik Gisvold

Norway

Thomas Glott

Norway

Ernestina Gomes

Portugal

Ping Gong

China

Katarina Göransson

Sweden

J. Carel Goslings

Netherlands

Colin Graham

Hong Kong

Gerrit Grieb

Germany

Raymond Grill

United States of America

Oliver Grottke

Germany

Carl Haasper

Germany

David Hak

United States of America

Peter Hallas

Denmark

Neil Hampson

United States of America

Sang Kuk Han

South Korea

Lauri Handolin

Finland

Jiandong Hao

United States of America

Katherine Harding

Australia

Kohei Hasegawa

United States of America

Dietrich Hasper

Germany

Bjorn Haug

Norway

Bjørn Olav Haugen

Norway

Matthias Helm

Germany

Eirik Helseth

Norway

Jon Kenneth Heltne

Norway

Elizabeth Henneman

United States of America

Johan Herlitz

Sweden

Manuel E. Herrera Gutierrez

Spain

Jochen Hinkelbein

Germany

Atsushi Hiraide

Japan

Frederik Hirsch

Germany

Ingrid Elise Hoff

Norway

John Holcomb

United States of America

Shaoping Hou

United States of America

Zhiyong Hou

China

Ives Hubloue

Belgium

Peter Hultén

Sweden

Steinar Hunskaar

Norway

Beverley Hunt

United Kingdom

Sissel Eikeland Husebø

Norway

Todd Huzar

United States of America

Per Kristian Hyldmo

Norway

Hideo Inaba

Japan

Joji Inamasu

Japan

Lenworth Jacobs

United States of America

Helena Jäntti

Finland

Hans Christian Jeske

Austria

Per Ingemar Johansson

Denmark

Jorine Juffermans

Netherlands

Afife Ayla Kabalak

Turkey

Sigridur Kalman

Sweden

Jeffry Kashuk

United States of America

Karl Kern

United States of America

Jan Kielstein

United States of America

Young

Min Kim – South Korea

Pål Klepstad

Norway

Claus Klingenberg

Norway

Marianne Klose

Denmark

Kai Knudsen

Sweden

Nobuaki Kobayashi

Japan

Alexander Koch

Germany

Nobuyasu Komasawa

Japan

Ulf Kongsgaard

Norway

Uwe Kreimeier

Germany

Thomas Kristiansen

Norway

Markku Kuisma

Finland

Jouni Kurola

Finland

Ching Huang Lai

Taiwan

Halvor J. B. Langeland

Norway

Alf Inge Larsen

Norway

Morten Schultz Larsen

Denmark

Kristoffer Lassen

Norway

Rifat Latifi

United States of America

Christian B Laursen

Denmark

Zeljko Lausevicn

Serbia

David Lawrence

United States of America

Abraham Layon

United States of America

Luke Leenen

Netherlands

Rolf Lefering

Germany

Ari Leppaniemi

Finland

Mehdi Lesko

United Kingdom

Being Chuan Lin

Taiwan

Joacim Linde

Sweden

Espen Lindholm

Norway

Thomas Lindner

Norway

Zhi Liu

China

John Lloyd

United Kingdom

Andrew Lockey

United Kingdom

Varut Lohsiriwat

Thailand

Ben Lovell

United Kingdom

Kare Lovstakken

Australia

Cathrine Lund

Norway

Thomas Lustenberger

Germany

Richard Lyon

United Kingdom

Dariusz Maciejewski

Poland

Stuart Marshall

Australia

Wenjun Martini

United States of America

Ingo Marzi

Germany

Gerald McGwin, Jr.

United States of America

Lauralyn McIntyre

Canada

Christian Medby

Norway

Hasse Melbye

Norway

Terje Meling

Norway

Kimberly Meyer

United States of America

Søren Mikkelsen

Denmark

Ole Christian Mjølstad

Norway

Nicolas Mongardon

France

Kevin Moran

New Zealand

Alessandro Morandi

Italy

Umberto Morelli

France

Rui Moreno

Portugal

Florentin Moser

Norway

Jarrod Mosier

United States of America

Pål Aksel Næss

Norway

Hiroyuki Nakao

Japan

Anders Rostrup Nakstad

Norway

Raj Nandi

United Kingdom

Giuseppe Nardi

Italy

Lais Navarro

Brazil

Saad Nseir

France

Mauro Oddo

Switzerland

Theresa Mariero Olasveengen

Norway

Henning Onarheim

Norway

Sisse R Ostrowski

Denmark

Jan Erik Otterstad

Norway

Nils Petter Oveland

Norway

Peter Paal

Austria

Cameron Palmer

Australia

Hyoung

chun Park – South Korea

Nick Parsons

United Kingdom

Giles Peek

United Kingdom

Tommaso Pellis

Italy

Søren Erik Pischke

Norway

Jerald Potts

Belgium

Cornelia Putz

Germany

Marius Rehn

Norway

Zaccaria Ricci

Italy

Louis Riddez

Sweden

Fred Rincon

United States of America

Kjetil G. Ringdal

Norway

Hannu Rintamaki

Finland

Brian Risavi

United States of America

Giuseppe Ristagno

United States of America

Leif Rognas

Denmark

Rainer Röhrig

Germany

Olav Røise

Norway

Louise Rose

Canada

Rolf Rossaint

Germany

Franco Ruberto

Italy

Vidar Ruddox

Norway

Soren Rudolph

Denmark

John Sakles

United States of America

Øyvind Salvesen

Norway

Mårten Sandberg

Norway

Sandro Scarpelini

Brazil

Herbert Schoechl

Austria

Inger Schipper

Netherlands

Kari Schirmer

Mikalsen

Hagen Schmal

Germany

Patrick Schober

Netherlands

Patrick Schoettker

Switzerland

David Seder

United States of America

Federico Semeraro

Italy

Serdar Sen

Turkey

Lalith Senarathna

Sri Lanka

Alex Senchenkov

United States of America

Tom Silfvast

Finland

Adam Singer

United States of America

Nils Kristian Skjaervold

Norway

David Sklar

United States of America

Eirik Skogvoll

Norway

Markus Skrifvars

Finland

Erik Sloth

Denmark

Anita Smith

United States of America

Wade Smith

United States of America

Curtis P Snook

United States of America

Stephen J M Sollid

Norway

Eldar Soreide

Norway

Endre Soreide

Norway

Philip Stahel

United States of America

Halvard Stave

Norway

Deborah Stein

United States of America

Christian Storm

Germany

Lovisa Strömmer

Sweden

Harry Sumnall

United Kingdom

Geir Arne Sunde

Norway

Kjetil Sunde

Norway

Leif Svensson

Sweden

Colman Taylor

Australia

Karim Tazarourte

France

Øyvind Thomassen

Norway

Julian Thompson

United Kingdom

Kenneth Thorsen

Norway

Ye Tian

China

Jens Tingleff

Denmark

Hideo Tohira

Australia

Else Tonnesen

Denmark

Satariya Trakulsrichai

Thailand

Håkon Trønnes

Norway

Chun

Hao Tsai

Natacha Turck

Switzerland

Oddvar Uleberg

Norway

Johan Unden

Sweden

Sonia Vaida

United States of America

Davide Liborio Vetrano

Italy

Wolfgang Voelckel

Austria

Giovanni Volpicelli

Italy

Erik von Elm

Switzerland

Christian Waage

Norway

Arasch Wafaisade

Germany

Dong Wang

United States of America

John Weigelt

United States of America

Mark Wilson

United Kingdom

Torben Wisborg

Norway

Cherylynn Wium

South Africa

Sebastian Wolfrum

Germany

Theodoros Xanthos

Greece

Youri Yordanov

France

Erik Zakariassen

Norway

Boris Zelle

United States of America

